# Altering Stomatal Density for Manipulating Transpiration and Photosynthetic Traits in Rice through CRISPR/Cas9 Mutagenesis

**DOI:** 10.3390/cimb45050245

**Published:** 2023-04-30

**Authors:** Sakthi Ambothi Rathnasamy, Rohit Kambale, Allimuthu Elangovan, Williams Mohanavel, Priyanka Shanmugavel, Gowtham Ramasamy, Senthil Alagarsamy, Rajavel Marimuthu, Veera Ranjani Rajagopalan, Sudha Manickam, Valarmathi Ramanathan, Raveendran Muthurajan, Geethalakshmi Vellingiri

**Affiliations:** 1 Department of Plant Biotechnology, Tamil Nadu Agricultural University, Coimbatore 641 003, Tamil Nadu, India; 2 Agro-Climatology Research Centre, Tamil Nadu Agricultural University, Coimbatore 641 003, Tamil Nadu, India; 3 Department of Crop Physiology, Tamil Nadu Agricultural University, Coimbatore 641 003, Tamil Nadu, India; 4 Sugarcane Breeding Institute, Coimbatore 641 007, Tamil Nadu, India

**Keywords:** rice, *OsEPF1*, stomatal density, photosynthetic efficiency

## Abstract

Stomata regulates conductance, transpiration and photosynthetic traits in plants. Increased stomatal density may contribute to enhanced water loss and thereby help improve the transpirational cooling process and mitigate the high temperature-induced yield losses. However, genetic manipulation of stomatal traits through conventional breeding still remains a challenge due to problems involved in phenotyping and the lack of suitable genetic materials. Recent advances in functional genomics in rice identified major effect genes determining stomatal traits, including its number and size. Widespread applications of CRISPR/Cas9 in creating targeted mutations paved the way for fine tuning the stomatal traits for enhancing climate resilience in crops. In the current study, attempts were made to create novel alleles of *OsEPF1* (Epidermal Patterning Factor), a negative regulator of stomatal frequency/density in a popular rice variety, ASD 16, using the CRISPR/Cas9 approach. Evaluation of 17 T_0_ progenies identified varying mutations (seven multiallelic, seven biallelic and three monoallelic mutations). T_0_ mutant lines showed a 3.7–44.3% increase in the stomatal density, and all the mutations were successfully inherited into the T_1_ generation. Evaluation of T_1_ progenies through sequencing identified three homozygous mutants for one bp insertion. Overall, T_1_ plants showed 54–95% increased stomatal density. The homozygous T_1_ lines (# E1-1-4, # E1-1-9 and # E1-1-11) showed significant increase in the stomatal conductance (60–65%), photosynthetic rate (14–31%) and the transpiration rate (58–62%) compared to the nontransgenic ASD 16. Results demonstrated that the genetic alterations in *OsEPF1* altered the stomatal density, stomatal conductance and photosynthetic efficiency in rice. Further experiments are needed to associate this technology with canopy cooling and high temperature tolerance.

## 1. Introduction

Global population is expected to cross 9.8 billion by 2050, which necessitates the doubling of food production. Being a staple cereal food crop, rice is consumed by more than 50% of the global population. Hence, increasing the production of rice plays a key role in achieving global food security [1]. Rice yield has undergone two major leaps in the past viz. first, during the 1960s, i.e., the green revolution when the semi-dwarfing gene was introduced, and secondly, through the introduction of hybrids during the 1980s [2,3]. Afterwards, no major advances have been reported in rice yield. Drought and high-temperature events are fast becoming major threats to increasing rice productivity under marginal environments. Any further increase in the rice yields must overcome major challenges viz. yield plateau, declining land, labour, water resources and finally, the increasing effects of climate change. The climate projections indicate an increased frequency in the occurrence of extreme weather events, thus affecting the rice yields significantly [4,5,6]. Hence, it has become important to develop the ‘climate-smart’ rice varieties that can alleviate the risk of crop failures due to extreme weather events [1,7,8].

Drought remains at the top in affecting rice productivity under both irrigated as well as rainfed conditions [6,9,10], followed by salinity [11,12,13] and flooding [14,15]. During recent years, high temperature has become a major yield-limiting factor, especially during flowering and grain-filling stages [16]. Substantial progress has been made in unravelling the genetic basis of drought tolerance traits and yield under drought conditions. However, only limited progress has been made in dissecting the physiological and genetic basis of high temperature tolerance traits in rice. A survey of literature indicated that apart from tissue tolerance and avoidance traits, canopy cooling may serve as a potential option to mitigate heat-induced yield losses. Genes involved in stomatal development and patterning have been functionally validated through transgenic and genome editing approaches [17,18,19,20,21]. Altering the stomatal density was proved to alter both stomatal conductance as well as CO_2_ assimilation rate, thus affecting the growth and metabolism of plants [17,22]. The genes belonging to the EPIDERMAL PATTERNING FACTOR-LIKE (EPFL) family (EPF1, EPF2 and EPFL9/STOMAGEN) encode for small secretory mobile peptides regulating stomatal development and its patterning [23]. As reported earlier, it is possible to alter the stomatal density by manipulating the expression patterns of EPF1, EPF2 and/or EPFL9 to attain the desired photosynthetic/transpiration traits [24,25,26]. In rice, the overexpression of the negative regulator *OsEPF1* reduces the stomatal density and the corresponding stomatal conductance [24,26]. Rice lines with reduced stomatal density exhibited enhanced water use efficiency and performed better under dry conditions [24,27]. On the other hand, the overexpression of the positive regulator *OsEPF9* increases the stomatal density [28], and knockout of *OsEPFL10* showed reduction in the stomatal density without any impact on stomatal conductance or carbon assimilation [27].

This may require development of a suitable genetic material harbouring increased/decreased stomatal distribution which can be used for analyzing the influence of stomatal traits on improving the photosynthetic efficiency and yield traits. Outcomes of evaluation revealed that the knockout mutants of *OsEPF1* showed increased stomatal density which, in turn, enhanced the stomatal conductance and photosynthetic efficiency.

## 2. Materials and Methods

### 2.1. Plant Materials

Seedlings of a popular rice variety ASD 16 were raised at Paddy Breeding Station, Tamil Nadu Agricultural University, Coimbatore, Tamil Nadu, India and transplanted after 21 days and maintained using prescribed procedures. Panicles were harvested at 12–15 days after flowering, and immature embryos were isolated and used for genetic transformation.

### 2.2. Construction of CRISPR/Cas9 Expression Vectors

Nucleotide sequence of *OsEPF1* (LOC_Os04g54490) was retrieved from the Ensembl Plants database (https://plants.ensembl.org/index.html (accessed on 27 December 2022)). The synthetic guide RNA that targets the region before the signal peptide cleavage site of the first exon (5′-GCTACTCTTGCTGACGCCCG-3′) was designed using the CRISPOR online tool (http://crispor.tefor.net/ (accessed on 27 December 2022)) [29]. The oligonucleotides were synthesized with adapter sequences 5′-GGCA-3′ attached to the forward primer and 5′-GGCA-3′ attached to the reverse primer so that the oligo-duplex can anneal and ligate with *Bsa*I digested pRGEB31 plasmid (Addgene plasmid # 51295) [30]. The pRGEB31 plasmid harbours guide RNA scaffold region and Cas9 gene, driven by Os U3 promoter and CaMV35S promoter, respectively. The oligonucleotides were annealed for duplex formation, and the sgRNA with sticky ends of *Bsa*I enzyme was cloned into *Bsa*I-digested pRGEB31 vector. The cloned sgRNA sequence was then confirmed by the Sanger sequencing method and mobilized into *Agrobacterium tumifaciens* strain LBA4404 using the freeze-thaw method [31]. *Agrobacterium* colonies were then screened using vector-specific primers (M13 reverse primer and Guide RNA reverse primer, Table 1) and used for transformation.

### 2.3. Agrobacterium-Mediated Transformation

Immature seeds dissected from the panicles harvested at 12–15 days after flowering were surface sterilized with 1.5% sodium hypochlorite along with a drop of Tween 20 for 3 min, followed by 70% ethanol treatment for 1 min and washed 4–5 times using sterile water. Isolated immature embryos were used for co-cultivation by the *Agrobacterium*-mediated transformation method [32]. Briefly, the isolated immature embryos were pretreated by incubating in a water bath at 42 °C for 30 min, followed by centrifugation at 1100× *g* for 10 min, after cooling the tube in ice for 1 min. Then, the embryos were transferred to the co-cultivation medium and infected with 5 µL of 3-day-old *Agrobacterium* culture. After co-cultivation, the proliferated calli were separated from the shoots and transferred to the first resting medium for 5 days and then to the second resting medium for 10 days. The calli proliferated in the resting medium were subjected to selection (twice) using 50 mg/L hygromycin and 250 mg/L cefotoxime. The selected calli were then regenerated using 1 mg/L NAA and 3 mg/L BAP with glutamine (30 mg/L). Then, the regenerated shoots were rooted in ½ MS medium containing 30 mg/L hygromycin. The putative transgenic plants were then hardened in green house facility available at the Centre for Plant Molecular Biology and Biotechnology, Tamil Nadu Agricultural University, Coimbatore.

### 2.4. Molecular Characterization of Transgenic Plants

The genomic DNA was isolated from both the transgenic and nontransgenic plants of ASD 16 using CTAB method [33] and used for PCR analysis. All the plants were screened using vector-specific primers (*hpt* forward primer and CaMV reverse primer), and the PCR-positive transgenic plants were analysed for the presence of mutation(s) through sequencing the target region. Table 1 shows a list of primers used for screening the transgenic plants and sequencing. The sequencing data of the target region was analysed using the ICE v3 CRISPR analysis tool (https://ice.synthego.com/ (accessed on 27 December 2022)) and DSDecode [34].

### 2.5. Measuring Stomatal Density and Stomatal Size

In order to measure the stomatal density, a fully expanded leaf was selected, and both abaxial and adaxial surfaces were cleaned using a tissue paper. Then, the stomatal impressions were made from the middle of the leaves using a transparent nail polish. Adequate care was taken to prepare impressions on both the sides of the midrib so as to avoid the bias in measuring the stomata number. After 10 min, the stomatal imprints were gently peeled off and immediately mounted on the glass slide. The number of stomata in both abaxial and adaxial surfaces of the leaf was counted using the images captured at 40× magnification, in a field of 0.2897 mm × 0.2184 mm = 0.06327 mm^−2^ using a fluorescence microscope (Radical RXLr-5, Ambala Cantt, India). The stomatal density (SD) was calculated using the formula: stomatal density = number of stomata/area in view. A total of ten fields of view were randomly observed in each impression. In each field view, a total of 10 stomata were randomly selected and used for recording the length and width of the stomata. Stomata size (SS) was calculated using the formula: SS = π × a × b, where a and b denote the semi-major and semi-minor axes, respectively.

### 2.6. Measurement of Gas Exchange Parameters

A portable photosynthesis analyser (LCpro SD, ADC BioScientific, Hoddesdon, UK) was used for the measurement of all the gas exchange parameters, such as the photosynthetic rate, stomatal conductance and the transpiration rate. All the measurements were recorded at the middle of the fully expended 3rd leaf. All the measurements were taken after the instrument reached the stability (2–10 min). The temperature maintained in the chamber was 28 °C, and the flow rate was 500 mL/min at a relative humidity of 60–65%. The CO_2_ concentration was 400 µmol mol^−1^ and the PPFD (Photosynthetic Photon Flux Density) was 1500 µmol m^−1^ s^−1^. The parameters measured are as follows: photosynthetic rate (A, µmol CO_2_ m^−2^ s^−1^), stomata conductance (gs mol H_2_O m^−2^ s^−1^) and transpiration rate (E, mmol H_2_O m^−2^ s^−1^). All the measurements were taken under light-saturated conditions. The water use efficiency was derived from the measured photosynthetic parameters. The ratio of photosynthetic CO_2_ assimilation (A) to transpiration rate (E) was calculated as the intrinsic water use efficiency.

### 2.7. Statistical Analysis

All of the required statistical analysis (ANOVA and DMRT) was performed using the R-Studio. Correlation analysis was also performed by using the R package by adopting the Pearson correlation method.

## 3. Results

### 3.1. Cloning of gRNA Targeting OsEPF1 and the Generation of Gene-Edited Rice Lines

**Figure 1:**
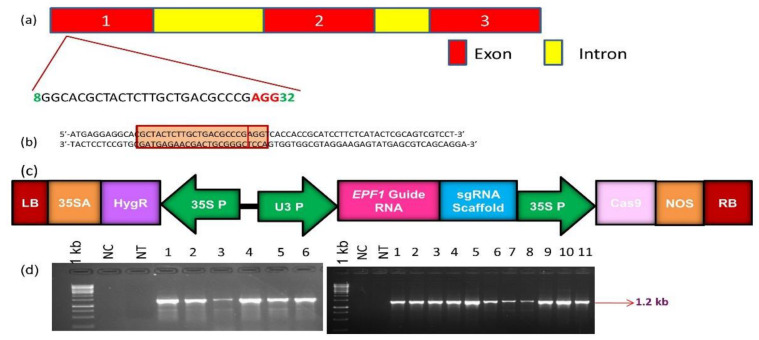
Development of the rice lines harboring mutations in *OsEPF1*: (**a**) Structure of *OsEPF1* (LOC_Os04g54490 on Chromosome 4) showing three exons and two introns; (**b**) The highlighted sequence shown in the red box represents the guide RNA, whereas the inner box represents the PAM; (**c**) T-DNA cassette of pRGEB31 harboring *OsEPF1* guide RNA. The expression of guide RNA scaffold is driven by OsU3 small nuclear RNA promoter; Cas9 gene is driven by CaMV35S promoter; hygromycin gene is driven by enhanced CaMV35S promoter; LB-left border RB-right border of T-DNA (**d**) PCR screening of transgenic plants (1–6 in the left panel and 1–11 in the right panel) using vector-specific primers (Table 1) 1 kb, DNA ladder, NC, negative control and \NT, Non transgenic ASD 16.

The nucleotide sequence, encoding *OsEPF1* (LOC_Os04g54490), was retrieved from the Ensembl Plants database. The *OsEPF1* gene contains three exons separated by an intron (Figure 1a). After the in silico evaluation of the target sequence, a small-guide RNA (sgRNA) targeting the exon1 of *OsEPF1* gene (LOC_Os04g54490) was designed using the CRISPOR online tool ((http://crispor.tefor.net/ (accessed on 27 December 2022)). The DNA oligos were synthesized along with forward (5′-GGCA-3′) and reverse (5′-AAAC-3′) adapter sequences (Figure 1a) and were cloned into pRGEB31 under the control of OsU3 promoter (Figure 1b,c). This plasmid was mobilized into *Agrobacterium* and used to transform the popular rice variety i.e., ASD 16.

About 175 immature embryos of the rice variety ASD 16 transformed using pRGEB31, harboring Cas9- *OsEPF1* gRNA, were subjected to two rounds of hygromycin selection. Putative transformed embryos survived after hygromycin selection were transferred to pre-regeneration followed by regeneration (refer to the Supplementary Figure S1). A total of 17 T_0_ progenies were regenerated, and PCR analysis using vector-specific and gene-specific primers revealed that all the 17 T_0_ lines were positive (Figure 1d).

### 3.2. Characterization of T_0_ Lines

**Figure 2:**
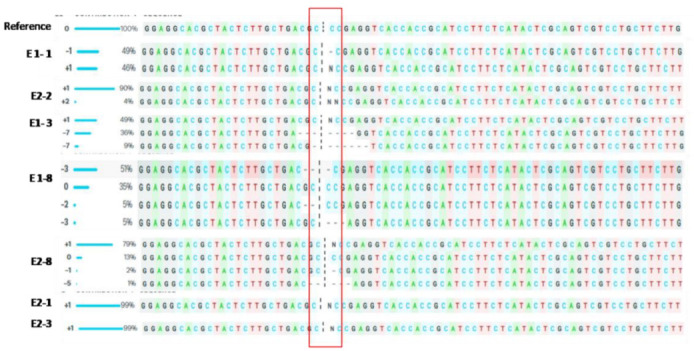
Sequence analysis of targeted region among the gene-edited lines (T_0_) using ICE CRISPR tool. Putative mutations were detected upon comparison against the reference sequence i.e., non-transgenic ASD 16 on the top. Sequence analysis of events E2-1 and E2-3 showed monoallelic mutation; E1-1 and E2-2 showed biallelic mutation, and E1-3, E1-8 and E2-18 showed multiallelic mutation. The vertical, black-dotted line represents the cut site of cas9 whereas the horizontal black hyphen denotes deletions and N indicates the insertion.

The genomic DNA, isolated from the gene-edited transgenic lines, were subjected to PCR amplification using primers flanking the target site, and the amplicons were subjected to Sanger sequencing. Non-transgenic (NT) ASD 16 was used as a reference. Outcomes of sequence analysis confirmed the presence of targeted mutation(s) in the 1st exon of *OsEPF1* across all the T_0_ lines (Figure 2). The ICE V2 CRISPR analysis identified seven multiallelic (E1-3, E 1-7, E 1-8, E1-9 and E2-4), seven biallelic (E1-1, E1-2, E1-4, E1-5, E1-6, E2-2 and E2-6) and three monoallelic mutations (E2-2, E2-3 and E2-5) (Table 2 and Supplementary Figure S2). T_0_ lines were found to harbour five different types of deletion mutations in the range of −1 bp to −7 bp and two types of insertion (+1 and +2 allele) mutations.

### 3.3. Phenotypic Evaluation of T_0_ Mutant Lines for Stomatal Density

**Figure 3:**
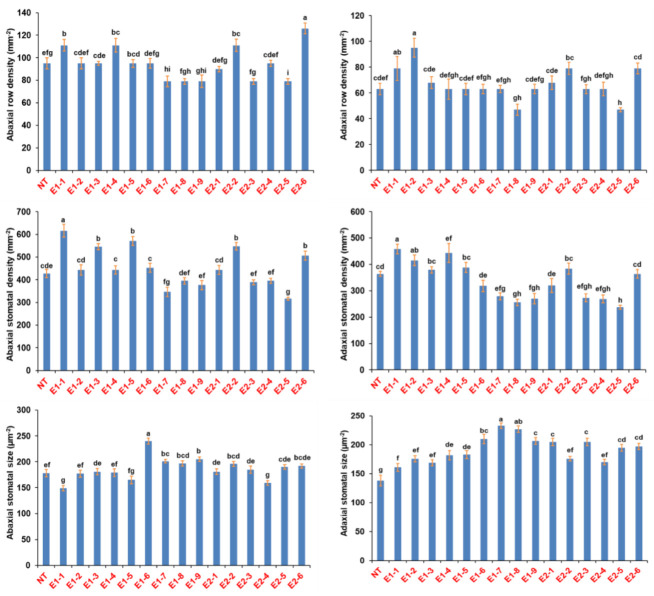
Adaxial and abaxial row density, stomatal density and stomatal size among the putative transgenic lines (T_0_) compared to the non-transgenic ASD 16 (NT). Gene edited lines showed altered stomatal parameters on both adaxial and abaxial side of leaves. Each data point represents an average of ten replications and error bar represents ± standard error of the mean data. Statistical significance was tested using Duncan’s multiple range test (DMRT). Different alphabets indicate the significance at *p* < 0.05.

All the 17 T_0_ lines were evaluated for stomatal density (SD) along with the nontransgenic ASD 16. Six lines showed significantly higher SD in both abaxial and adaxial surface than the nontransgenic ASD 16 (Figure 3). The line #E1-1 showed maximum (44.3%) increase in the abaxial SD followed by the lines viz. E1-5, E2-2, E1-3, E2-6, E1-6, E1-2, E2-1 and E1-4 with a 3.7 to 33.7% increase. Overall, the stomatal density of the adaxial leaf surface was found to be lower than the abaxial surface. The mutant line #E1-1 showed significantly higher abaxial and adaxial stomatal density compared to the nontransgenic ASD 16. Mutant lines #E1-7, E1-8, and E2-5 have displayed reduced stomatal density accompanied by decreased row density and increased stomatal size.

### 3.4. Phenotypic Evaluation of T_1_ Progenies

**Figure 4:**
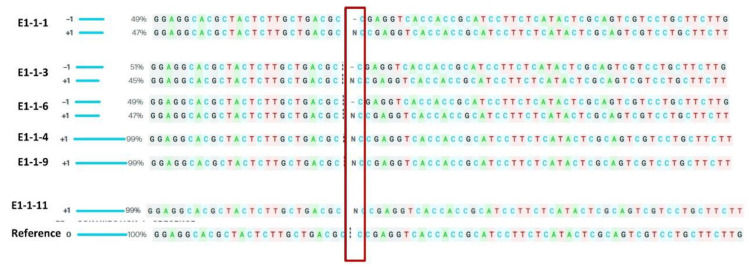
Sequence analysis of the target region among the T_1_ progenies of # E1-1 using ICE CRISPR tool. Mutants #E1-1-4, E1-1-9, E1-1-11 showed homozygous monoallelic mutation and the progenies E1-1-1, E1-1-3, E1-1-6 showed biallelic mutation on sequence analysis. The vertical, black-dotted line represents the cut site of cas9 whereas the horizontal black hyphen denotes the deletions and N indicates the insertion.

The inheritance pattern of the altered stomatal traits was studied in the T_1_ generation. The mutant line #E1-1 harbouring a biallelic mutation (+1/−1) and significantly higher stomatal density in the T_0_ generation was forwarded to the T_1_ generation. Six progenies (# E1-1-1, # E1-1-3, # E1-1-4, # E1-1-6, # E1-1-9 and # E1-1-11) were analysed against the non-transgenic ASD 16. Sequence analysis identified three lines, namely, E1-1-4, #E1-1-9 and #E1-1-11 showing homozygous mutation (+1 bp insertion) and remaining three mutant lines (#E1-1-1, #E1-1-3 and #E1-1-6) showing the mutation(s) under heterozygous mutation (+1/−1) (Figure 4; Supplementary Figure S3).

**Figure 5:**
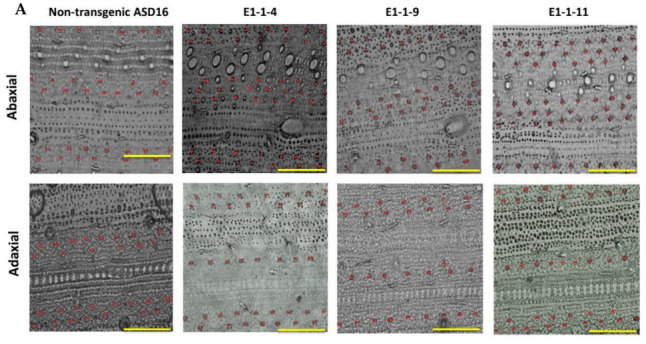

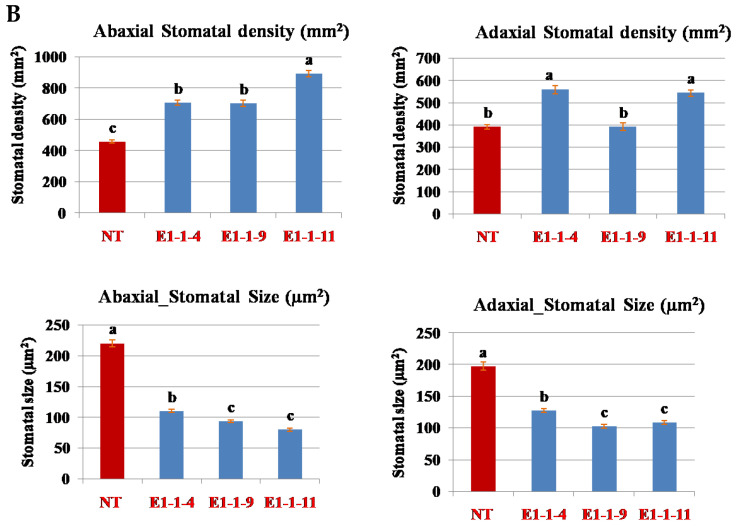
*OsEPF1* edited mutants showed altered stomatal density and stomatal size. (**A**). Stomatal distribution in the T_1_ progenies of *OsEPF1* edited lines against its wild type ASD 16. Homozygous mutants showed increased stomatal density and altered stomatal file density compared to non-transgenic ASD 16. The clustered stomata showed reduction in stomatal size. The top row represents the stomatal density in abaxial side, whereas the bottom row represents the stomatal density in adaxial leaf surface. Scale bar = 100 µm. (**B**). Graphical representation of stomatal density and stomatal size in the T_1_ transgenic lines compared against the non-transgenic ASD 16 (NT). Each data point represents an average of 10 replications and error bar represents ± standard error of the mean data. Statistical significance was tested using Duncan’s multiple range test (DMRT). Different alphabets indicate the significance at *p* < 0.05.

All the three homozygous T_1_ mutant lines i.e., #E1-1-4, #E1-1-9 and #E1-1-11 were found to possess significantly higher number of stomata (Figure 5). The line #E1-1-11 was found to possess 95% increased stomatal density in the abaxial surface than the nontransgenic ASD 16. This increase in the stomatal density was accompanied by a corresponding reduction in the stomatal size. Mutant lines showed a 50–64% reduction in the size of stomata in the abaxial surface and 35–48% reduction in the size of stomata in the adaxial surface (Table 3). Mutants had an increased number of stomatal files leading to increased stomatal density.

### 3.5. Physiological Evaluation of the T_1_ Mutant Lines

**Figure 6:**
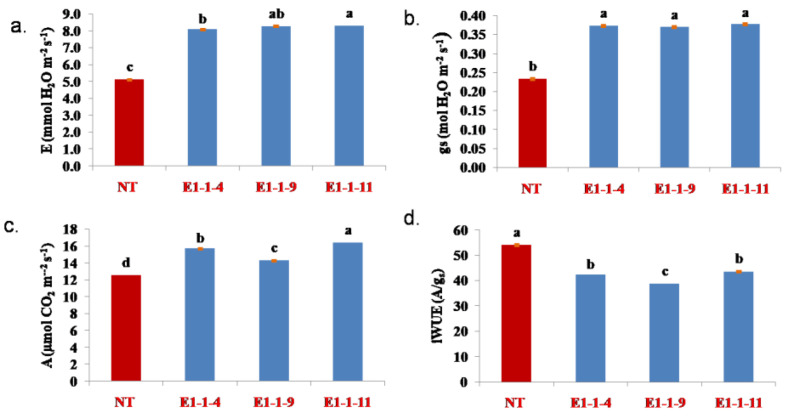
Evaluation of the homozygous mutant lines for stomatal conductance, photosynthetic rate and transpiration rate. NT, nontransgenic ASD 16; #E1-1-4, E1-1-9 and #E1-1-11 represented the *OsEPF1* gene edited lines. (**a**). transpiration rate; (**b**). stomatal conductance, (**c**). carbon dioxide assimilation rate (**d**). water use efficiency (A/gs). All values of stomatal density and stomatal size are mean ± standard error; each value represents the mean of ten replications and different letters indicates significance at *p* < 0.05 compared.

**Figure 7:**
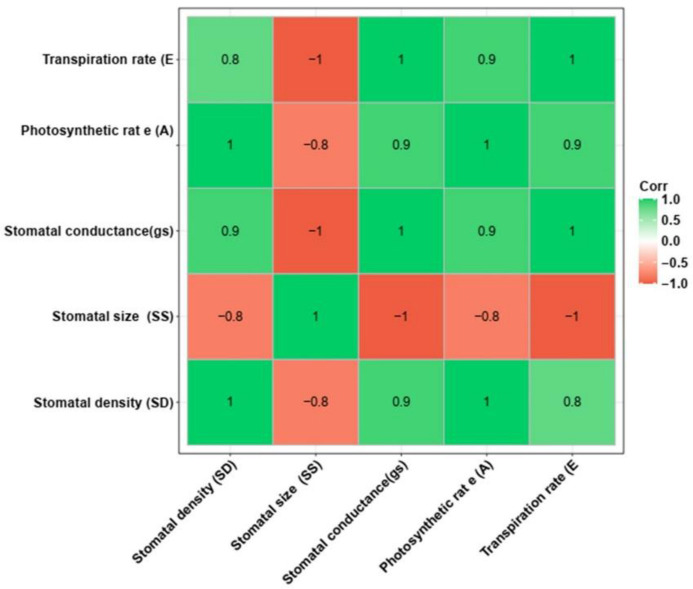
Correlation analysis between the stomatal traits and physiological parameters. Stomatal density showed positive correlation with stomatal conductance, photosynthetic rate and transpiration rate and negative correlation to stomatal size.

The T_1_ mutant progenies (#E1-1-4, #E1-1-9 and #E1-1-11) harbouring homozygous mutation (+1 bp) were evaluated for stomatal conductance (g_s_), photosynthetic rate (A) and the transpiration rate (E) (Figure 6). The mutant (T_1_) lines showed a 60–65% increased stomatal conductance than the nontransgenic ASD 16. The line #E1-1-11 recorded the maximum increase in its stomatal conductance (65.2%) and photosynthetic rate (31%) over the nontransgenic ASD 16, followed by E1-1-4 and E1-1-9. Similarly, the transpiration rate was also found to be significantly higher in all three T_1_ progenies i.e., 58–62% higher transpiration rate than NT ASD 16. The correlation analysis results showed a significant positive association between stomatal density and other parameters, such as g_s_, A and E. Further, the stomatal density was found to be negatively correlated with stomatal size (Figure 7). The mutants showed 20–29% decrease in water use efficiency compared to nontransgenic ASD 16.

## 4. Discussion

Extreme weather events viz. drought vs. flood and high temperature vs. cold stresses are predicted to occur at a high frequency in the coming years due to rapidly changing climatic conditions. Genetic improvement of the staple cereal rice for enhanced tolerance to heat and drought stresses will help in sustaining rice production under future climatic conditions. Maintenance of water relations between the plant and soil is vital for the regulation of plants’ responses to both drought and heat stresses. Stomata plays a vital role in regulating gas and water exchange in plants by facilitating the physiological processes, such as the transpiration, stomatal conductance and photosynthesis [35]. Precise genetic manipulation of the stomatal traits may help in altering the crops’ responses to drought and high temperature. Any increase in the stomatal density enhances the stomatal conductance (gs) which, in turn, favours high evaporative cooling to overcome heat-induced damages [20,36,37]. This warrants a thorough understanding of the molecular events regulating stomatal traits, such as the stomatal files, density and size. At present, the knowledge generated from the species *Arabidopsis thaliana* is utilized for the genetic improvement of stomatal traits in other grass species [26,38,39,40]. Several investigations have been conducted to alter the stomatal traits (density and size) through functional genomics approaches. The EPIDERMAL PATTERNING FACTOR and EPIDERMAL PATTERNING FACTOR (LIKE) family of genes (*EPF1*, *EPF2* and *EPFL9*/STOMAGEN) were found to regulate the stomatal density [24,25,26,27,28,41,42]. The effect of altered stomatal density on the photosynthetic traits was shown to depend on plant species and the environmental conditions [43,44,45]. The transgenic *Arabidopsis thaliana* plants with reduced stomatal density exhibited a reduction in leaf transpiration. On the other hand, the *epf1*/*epf2* knock out mutants with increased stomatal density exhibited a high transpiration rate [22]. Plants with an increased transpiration rate recorded a reduction in leaf temperature than their controls. This revealed the role of stomatal density in regulating transpiration rate [22]. Generally, the plants with increased stomatal density exhibit enhanced stomatal conductance, carbon dioxide assimilation and photosynthetic rate [17,18]. Sakoda, et al. [42] attempted to manipulate the CO_2_ diffusion by altering the stomatal density so as to enhance the photosynthetic capacity in plants, using *EPF1* knockout mutants. Several other studies have also revealed that the stomatal density is positively correlated with photosynthetic capacity and stomatal conductance [46,47]. The PATROL1 over-expression line with faster stomatal opening responses and the slac1 (slow anion channel-associated 1) and ost1 (open stomata 1) mutants with stay-open stomata all displayed greater photosynthetic rates and growth rate under variable light conditions than the wild type [48]. The improvement of stomatal response in fluctuating light conditions in a natural environment will greatly improve photosynthesis rate and yield.

In the current study, the authors used CRISPR/Cas9 approach to alter the stomatal density of a lowland-irrigated rice variety, ASD 16, through targeted suppression of *OsEPF1*. The immature embryos of ASD 16 were genetically transformed using *OsEPF1* gRNA harbouring a genome editing vector which produced a total of 17 mutant progenies. The sequencing analysis results of 17 T_0_ lines identified seven progenies with multi-allelic mutations, seven with biallelic and three with monoallelic mutations. The T_0_ lines showed significant increase (3.7–44.3%) in their stomatal density compared to the nontransgenic ASD 16. The mutant line # E1-1, harboring a biallelic mutation (+1/−1), showed a maximum of 44.3% increase in its stomatal density. The mutants E1-7, E-8, and E2-5 showed increased stomatal size and decreased row density.

As per the previous reports, altering expression of *EPF1*, *EPF2* and *EPFL9* was reported to alter the stomatal density [24,25,26,42] and water use efficiency [27]. *EPF1* and *EPF2* were reported to function as negative regulators of the stomatal development, whereas *EPFL9* was shown to be a positive regulator [41,42]. The *Arabidopsis* lines that over-express the STOMAGEN/*EPFL9*- line (ST-OX) showed a 268.1% increase in the stomatal density [42]. The EPIDERMAL PATTERNING FACTOR 1 knockout line (*epf1*) of *Arabidopsis* showed a 46.5% increased stomatal density than the wild type [42].

The inheritance of mutation and its effect on stomatal characteristics was assessed in the T_1_ generation. In the current study, evaluation of T1 progenies of the T_0_ line # E1-1 identified three progenies, namely, # E1-1-4, # E1-1-9 and # E1-1-11 possessing +1 bp mutation under the homozygous condition. The mutant line E1-1-11 showed 95% increase in stomatal density. The homozygous mutants showed increased stomatal file density compared to nontransgenic ASD 16.

The homozygous progenies showed a greater increase in stomatal conductance (gs), photosynthetic rate (A) and transpiration rate (E) than the nontransgenic ASD 16. The mutant progenies showed a 60–65% increase in their stomatal conductance, 14–31% increase in their photosynthetic rate and a 58–62% increase in their transpiration rate. Among staple crops, rice is reported to possess small-sized stomata with high density [36]. The transgenic plants of IR 64, over-expressing *OsEPF1*, showed an increase in the canopy temperature (0.3 °C warmer) under adequately irrigated conditions [24]. The speed at which the stomata can respond to the changing environment is important to improve the short-term water use efficiency [36,49,50]. Pitaloka et al. [50] inferred that the small-sized stomata respond much faster than the larger ones.

An *Arabidopsis* mutant with increased stomatal density showed a 30% higher CO_2_ assimilation rate than the wild type under high light intensities [18]. A similar condition is predicted under elevated CO_2_ levels with decreased stomatal conductance since it limits the CO_2_ fixation rate [51]. However, the crops with high density of stomata possess high gas exchange potential, an important trait to mitigate the effects of heat stress by transpiration-mediated cooling. Poor stomatal conductance may be a major cause of photosynthetic limitation. In order to overcome the insufficient photosynthesis as a result of low stomatal density, genome engineering may pave the way to increase the stomatal density by improving the stomatal conductance, photosynthetic efficiency as well as biomass production in field crops. In the present study, we have attempted to increase the stomatal conductance of rice by altering the stomatal density. The lines generated in the study will be further evaluated for their responses to high temperature stress conditions; hence, tolerant lines under diverse environmental conditions will be identified for future crop improvement programmes.

## Figures and Tables

**Table 1:** List of the primers used in the study.

Name of the Construct/Organism Name of the Gene/	Selection Marker	Forward and Reverse Primers	Annealing Temperature	Size of the Amplicon
*Agrobacterium tumefaciens* harboring pRGEB31-*EPF1*-sgRNA	*CaMV35S* and *hpt*	hpt–F: 5′ TACACAGCCATCGGTCCA 3′	59 °C	1.3 kb
		CaMV35S -R: 5′ ACCTCCTCGGATTCCATTGC 3′		
*E*. *coli* harboring pRGEB31-*EPF1*-sgRNA	*M13* and *EPFs* gRNA	*M13* -R: 5′ TCACACAGGAAACAGCTATG 3′	55 °C	445 bp
		*EPF* sgRNA *R*-AAACCGGGCGTCAGCAAGAGTAGCG		
*Oryza sativa* (ASD 16)	*CaMV35S* and *hpt*	hpt–F: 5′ TACACAGCCATCGGTCCA 3′ 59 °C 1.3 kb	59 °C	1.3 kb
		CaMV35S -R: 5′ ACCTCCTCGGATTCCATTGC 3′		
	Gene specific primer *EPF1*	EPF F- CAATGGCTGCACACACATATAC	58 °C	400 bp
		EPFR-CAAGCAAGCACATTCGGTAAG		

**Table 2:** Mutations observed in the *OsEPF1* gene-edited transgenic lines (T_0_) of ASD 16.

Line No	Mutation Type	Mutation Allele Size
E1-1	Biallelic	+1, −1
E1-2	Biallelic	+1, −1
E1-3	Multiallelic	+1, −7, −7
E1-4	Biallelic	+1, −1
E1-5	Biallelic	+1, −1
E1-6	Biallelic	+1, −1
E1-7	Multiallelic	−3, 0, −2, −3
E1-8	Multiallelic	−3, 0, −2, −3
E1-9	Triallelic	+1, −1, 0
E2-1	Monoallelic	+1
E2-2	Biallelic	+1, +2
E2-3	Monoallelic	+1
E2-4	Triallelic	+1, −1, 0
E2-5	Monoallelic	+1
E2-6	Biallelic	+1, −1
E2-7	Multiallelic	+1, −1, 0, −5
E2-8	Multiallelic	+1, −1, 0, −5

**Table 3:** Stomatal density and percent increase in adaxial and abaxial stomatal densities of the homozygous T_1_ progenies of *OsEPF1* gene-edited plants compared against the nontransgenic ASD 16. Data of stomatal density and stomatal size represented as ±standard error; each value represents the mean of 10 replications and different letters indicate the significance at *p* < 0.05.

Event	Abx_SD_mm	(%) Increase in Abaxial_SD (mm^−2^)	Adx_SD_mm	(%) Increase in Adaxial_SD (mm^−2^)	Abaxial Stomatal Size (µm^2^)	Adaxial Stomatal Size (µm^2^)
NTL	456 ± 10.7 ^c^	-	391 ± 9.98 ^b^	-	219.8 ± 5.37 ^a^	197.2 ± 6.6 ^a^
E1-1-4	706 ± 16.11 ^b^	54	557 ± 19.14 ^a^	42.5	109.9 ± 2.53 ^b^	127.1 ± 2.8 ^b^
E1-1-9	701 ± 19.82 ^b^	53.7	391 ± 17.02 ^b^	0	93.2 ± 2.18 ^c^	102.6 ± 2.78 ^c^
E1-1-11	891 ± 21.26 ^a^	95	543 ± 14.29 ^a^	38.8	79.5 ± 2.47 ^c^	108.2 ± 2.98 ^c^

## Data Availability

Not applicable.

## Supplementary Materials

The following supporting information can be downloaded at: https://www.mdpi.com/article/10.3390/cimb45050245/s1, Figure S1. *Agrobacterium*-mediated transformation of immature embryos of rice cultivar ASD16 with pRGEB31 vector harbouring *OsEPF1*-sgRNA. (a) Co-cultivated immature embryos in co-cultivation media; (b) Immature embryo derived calli after 7 days of co-cultivation; (c and d) Calli at first and second resting stage; (e) Calli at selection stage after 32 days of co-cultivation; (f) Calli at shoot regeneration satge; (g) Regenerated shoots under first rooting; (h) Second stage rooting; (i) Hardening of putative transgenic plants in portrays; Figure S2. Chromatograms of T0 *OsEPF1* gene edited lines: Sequencing results (chromatogram) showing the altered sequence at the target region in the genome edited lines compared to the non-transgenic control. For each sample the top chromatogram is of the edited line and the bottom one is of non-transgenic. The horizontal black underline represents the guide RNA sequence. The horizontal red underline indicates the PAM site. The vertical black dotted line represents the mutated site which is also highlighted using vertical red box; Figure S3. Sanger sequencing analysis of T_1_ lines: Sequencing results (chromatogram) showing the altered sequence at the target region in the genome edited lines compared to the non-transgenic control. For each sample the top chromatogram is of the edited line and the bottom one is of non-transgenic. Homozygous mutant lines showed insertion (+1 bp) at 3 bp upstream of PAM site. The horizontal black underlined region represents the guide RNA sequence. The horizontal red underline is the PAM site. The vertical black dotted line represents the mutated site. Red box indicates the insertions (T). In each black rectangle, bottom lane represents the wild allele and the upper lane represents the corresponding edited line.
