# Supplementary material for: Altering Stomatal Density for Manipulating Transpiration and Photosynthetic Traits in Rice through CRISPR/Cas9 Mutagenesis
Source: Curr Issues Mol Biol. 2023 Apr 30;45(5):3801–14. doi: 10.3390/cimb45050245 (PMC10217023; doi:10.3390/cimb45050245)
Supplement: Supplementary file 1 [file cimb-45-00245-s001.zip › cimb-2156215-supplementary.pdf]

Supplementary figures:

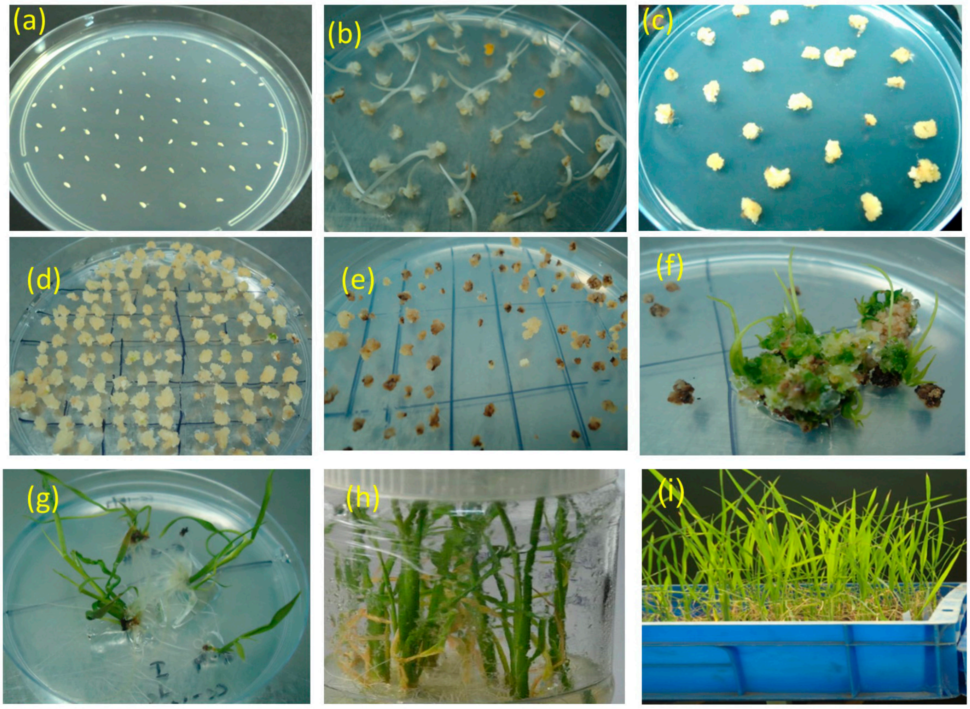

Supplementary Figure 1. *Agrobacterium*-mediated transformation of immature embryos of rice cultivar ASD16 with pRGEB31 vector harbouring *OsEPF1*-sgRNA. (a) Co-cultivated immature embryos in co-cultivation media; (b) Immature embryo derived calli after 7 days of co-cultivation; (c and d) Calli at first and second resting stage; (e) Calli at selection stage after 32 days of co-cultivation; (f) Calli at shoot regeneration satge; (g) Regenerated shoots under first rooting; (h) Second stage rooting; (i) Hardening of putative transgenic plants in portrays

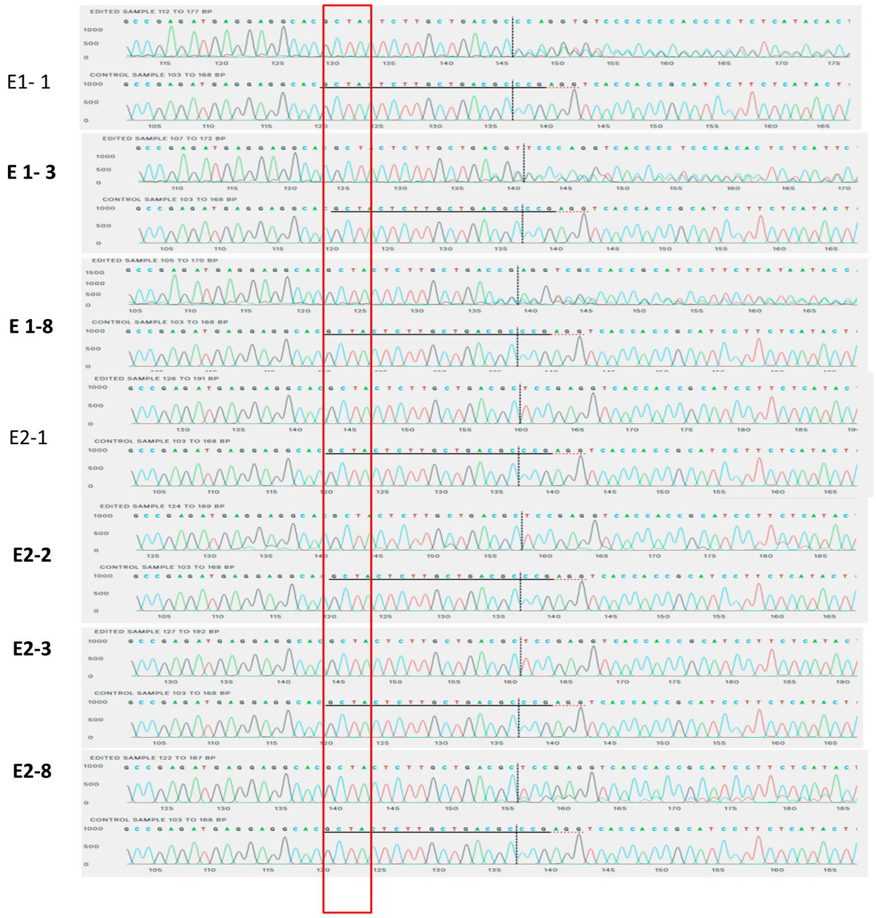

Supplementary Figure 2. Chromatograms of T0 *OsEPF1* gene edited lines: Sequencing results (chromatogram) showing the altered sequence at the target region in the genome edited lines compared to the non-transgenic control. For each sample the top chromatogram is of the edited line and the bottom one is of non-transgenic. The horizontal black underline represents the guide RNA sequence. The horizontal red underline indicates the PAM site. The vertical black dotted line represents the mutated site which is also highlighted using vertical red box.

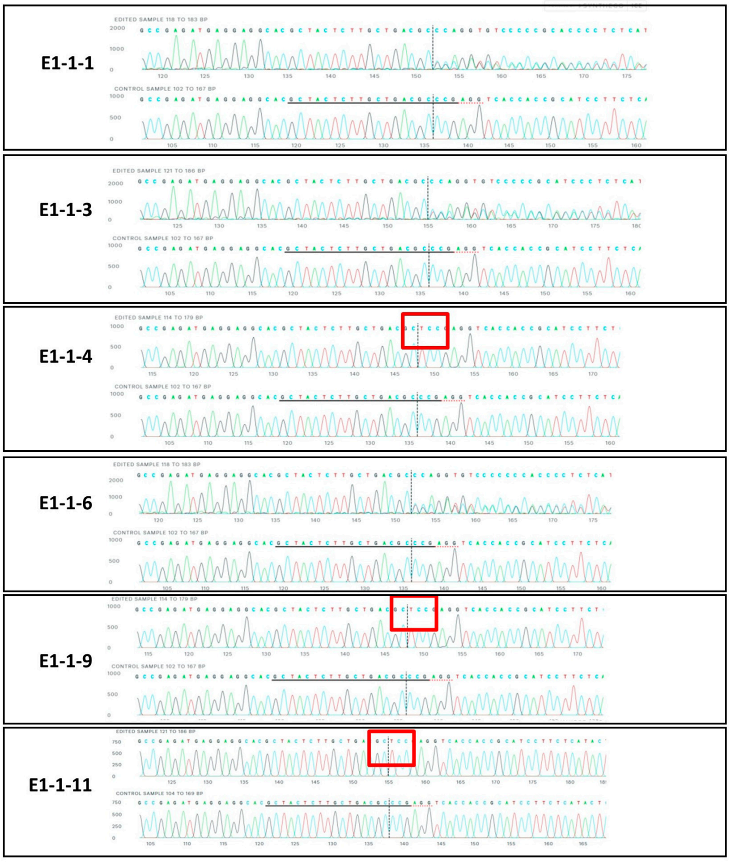

Supplementary Figure 3. Sanger sequencing analysis of T_1 lines: Sequencing results (chromatogram) showing the altered sequence at the target region in the genome edited lines compared to the non-transgenic control. For each sample the top chromatogram is of the edited line and the bottom one is of non-transgenic. Homozygous mutant lines showed insertion (+1 bp) at 3 bp upstream of PAM site. The horizontal black underlined region represents the guide RNA sequence. The horizontal red underline is the PAM site. The vertical black dotted line represents the mutated site. Red box indicates the insertions (T). In each black rectangle, bottom lane represents the wild allele and the upper lane represents the corresponding edited line.
